# The Incremental Prognostic Value of Hepatocyte Growth Factor in First-Ever Acute Ischemic Stroke: An Early Link Between Growth Factor and Interleukins

**DOI:** 10.3389/fneur.2021.691886

**Published:** 2021-08-04

**Authors:** Fangfang Li, Ping Liu, Yuyou Huang, Lingzhi Li, Sijia Zhang, Zhenhong Yang, Rongliang Wang, Zhen Tao, Ziping Han, Junfen Fan, Yangmin Zheng, Haiping Zhao, Yumin Luo

**Affiliations:** ^1^Institute of Cerebrovascular Diseases Research and Department of Neurology, Xuanwu Hospital of Capital Medical University, Beijing, China; ^2^National Clinical Research Center for Geriatric Disorders, Beijing, China; ^3^Beijing Institute for Brain Disorders, Beijing, China

**Keywords:** acute ischemic stroke, HGF, IL-16, neutrophil, prognosis

## Abstract

Hepatocyte growth factor (HGF) is a potential prognostic factor for acute ischemic stroke (AIS). In this study, we sought to validate its earlier predictive accuracy within 24 h for first-ever AIS. Moreover, as HGF interacts with interleukins, their associations may lead to novel immunomodulatory therapeutic strategies. Patients with first-ever AIS (*n* = 202) within 24 h were recruited. Plasma HGF and related interleukin concentrations were measured by multiplex immunoassays. The primary and secondary outcomes were major disability (modified Rankin scale score ≥3) at 3 months after AIS and death, respectively. Elastic net regression was applied to screen variables associated with stroke outcome; binary multivariable logistic analysis was then used to explore the relationship between HGF level and stroke outcome. After multivariate adjustment, upregulated HGF levels were associated with an increased risk of the primary outcome (odds ratio, 7.606; 95% confidence interval, 3.090–18.726; *p* < 0.001). Adding HGF to conventional risk factors significantly improved the predictive power for unfavorable outcomes (continuous net reclassification improvement 37.13%, *p* < 0.001; integrated discrimination improvement 8.71%, *p* < 0.001). The area under the receiver operating characteristic curve value of the traditional model was 0.8896 and reached 0.9210 when HGF was introduced into the model. An elevated HGF level may also be a risk factor for mortality within 3 months poststroke. The HGF level was also positively correlated with IL-10 and IL-16 levels, and HGF before interaction with all interleukins was markedly negatively correlated with the lymphocyte/neutrophil ratio. HGF within 24 h may have prognostic potential for AIS. Our findings reinforce the link between HGF and interleukins.

## Introduction

Ischemic stroke is a prevalent disease with high disability and mortality. The current treatments for acute ischemic stroke (AIS) are intravenous administration of tissue plasminogen activator (t-PA) and endovascular treatment to recanalize the blood flow. The prediction of clinical outcomes after AIS is increasingly accepted by physicians in several steps of stroke evaluation for acute therapies, palliative care, or rehabilitation (1, 2). However, traditional risk factors are not comprehensive enough to predict the prognosis of AIS patients. Therefore, the accurate identification of novel biomarkers to improve risk stratification in patients with ischemic stroke is desirable to aid in making decisions regarding stroke care and management.

As hepatocyte growth factor (HGF) is present in the circulation after endothelial injury, its higher levels correlate with multiple cardio-cerebrovascular diseases, including atherosclerosis (3), diabetes mellitus (3), and acute myocardial infarction (4). HGF has received attention as a potential biomarker of AIS. First, circulating HGF levels can be referred to predict the risk of ischemic stroke (3). Second, HGF levels may be useful in diagnosing ischemic stroke as an earlier study shows that serum levels of HGF in patients with cerebral infarction are significantly increased during the early stage and remain elevated until 7 d poststroke and that higher HGF concentrations are correlated with lower gains in the Stroke Impairment Assessment Set in stroke rehabilitation. Recently, serum HGF levels were shown to be associated with poor prognosis at 3 months independent of stroke severity, especially in patients with AIS without heparin pretreatment (5). HGF was also independently associated with death and major disability in patients with AIS with dyslipidemia (6). However, these studies excluded patients undergoing anticoagulation therapy because heparin has antifibrotic activity, mediated by the cellular secretion of HGF (7), and HGF is activated by t-PA (8), which abates its prognostic value. In addition, the patients in the above studies were recruited within 48 h of symptom onset; however, HGF level is already increased within 24 h (9).

The causal role of HGF in cerebral vascular disease has not been fully elucidated. Recent research points to a link between HGF level and phenotypic transformation of immune cells. A link between HGF levels and basal metabolic rate was mediated by IL-16 in patients with obesity-related nonalcoholic fatty liver disease (10). In addition, HGF inhibited microglia activation and the expression of pro-inflammatory IL-1β in a rat model of cerebral ischemia (11); it also promoted M2 macrophage transition and the expression of anti-inflammatory IL-10 and facilitated muscle regeneration (12). Mesenchymal stem cell–secreted HGF contributed to the conversion of fully differentiated Th17 cells into functional T regulatory (Treg) cells (13); however, HGF decreased the levels of Th2 cytokines (IL-4, IL-5, and IL-13) in bronchoalveolar lavage fluid and attenuated airway hyperresponsiveness (14). Although interleukin levels (IL-16, IL-1β, IL-5, and IL-10) are detected in patients with AIS, their prognostic value or relationship with HGF is unknown. Therefore, the prognostic value of HGF within 24 h after stroke with functional outcome (modified Rankin scale and death at 90 d) in patients with first-ever AIS with or without heparin/t-PA treatment requires validation. Understanding the associations of the plasma HGF level and its related interleukins in clinical populations in the acute phase may offer novel immunomodulatory therapeutic options.

## Materials and Methods

### Study Participants and Outcome Assessment

In the present study, we analyzed consecutive patients with first-ever AIS who presented to Xuanwu Hospital of Capital Medical University within 24 h after symptom onset between November 2018 and May 2019. Our study was approved by the Ethics Committee of Xuanwu Hospital, Capital Medical University. Written informed consent was provided by all patients or their immediate family members. According to the criteria of the Trial of Org 10,172 in Acute Stroke Treatment (TOAST), ischemic stroke is classified as large artery atherosclerosis (thrombotic), cardiac embolism (embolic), or small artery occlusion lacunae (lacunar). The inclusion criteria were (1) patients with focal or global neurological deficits, (2) brain computed tomography (CT) or magnetic resonance imaging (MRI) findings indicating a diagnosis of AIS, (3) clinical evaluation performed and recorded 90 d after stroke, and (4) no previous history of stroke. Patients with heart failure, renal failure, cancer, immune diseases, active infection, rheumatic heart disease, liver cirrhosis, epilepsy, and other neurological diseases as well as serious pancreas, intestine, thyroid, or lung disease were excluded from the present study. Finally, 202 patients with first-ever AIS were included for analysis. The primary outcome was defined as an unfavorable outcome [modified Rankin scale (mRS) score, 3–6] at 90 d after stroke, and the secondary outcome was death within 3 months after stroke. The follow-up was conducted by experienced neurologists blinded to the experimental design.

### Clinical Data and Blood Collection

Baseline data on demographic characteristics; clinical features, including onset time and systolic and diastolic pressure; medical history; and routine laboratory examination (leukocyte number, glucose levels, blood lipids, etc.) at admission were recorded from the electronic medical record system. Stroke severity was evaluated using the NIH Stroke Scale (NIHSS) score by experienced neurologists at admission (15). Blood samples were collected from patients with AIS before they received any treatments. The blood samples were centrifuged at 3,000 rpm for 10 min to obtain plasma. All plasma samples were frozen at −80°C before the examination.

### Measurement of Circulating Biomarker Levels

Plasma levels of HGF, IL-1β, IL-10, IL-5, and IL-16 were examined using a ProcartaPlex multiplex magnetic bead panel kit (Invitrogen, PPX-10) according to the manufacturer's protocol. The data were analyzed using ProcartaPlex Analyst 1.0 software. The experienced laboratory technicians who conducted the tests were blinded to the experimental design.

### Statistical Analysis

Data were analyzed using IBM SPSS Statistics for Windows, version 21.0 (IBM Corp., Armonk, NY, USA) and R software (version 3.5.1), and *p* < 0.05 was defined as statistically significant. Continuous variables are expressed as the means ± standard deviations (SDs) and analyzed using Student's *t*-test (normal distribution) or medians with interquartile ranges (IQRs) and analyzed using Mann–Whitney U tests (nonnormal distribution). Frequencies and percentages in categorical variables were analyzed by chi-squared tests. Receiver operating characteristic (ROC) curves were generated to identify the predictive values of the five cytokines in predicting AIS and calculating the optimal cutoff values of HGF and the four inflammatory biomarkers. An elastic net regression model was used to select variables that were potentially related to stroke outcome with cross-validation applied to determine the optimal regularization parameters (16, 17). In logistic regression models that introduced penalty terms, variables with estimated coefficients close to zero were removed from the model for variable selection. Finally, seven variables were selected, and their corresponding odds ratios (ORs) and 95% confidence intervals (CIs) were reported. We considered variables with *p* < 0.05 to be statistically significant. The five significant variables selected from the elastic net regression model were then imported into the binary multivariable stepwise logistic regression analysis to explore the relationship between HGF level and stroke outcome.

Spearman rank correlation analysis was used to explore the relationship between HGF and other factors. Finally, net reclassification improvement (NRI) and integrated discrimination improvement (IDI) (18) were used to evaluate whether adding one or more inflammatory biomarkers to conventional risk factors improved the predictive power for the primary and secondary outcomes in patients with AIS.

## Results

### Baseline Characteristics

The baseline clinical characteristics of the 202 patients with AIS are listed in Table 1. The mean patient age was 64.24 years, and 149 (73.76%) of the patients were male. The median NIHSS score at presentation was 5 (IQR 3–11), and the median time from stroke onset to blood collection was 2.95 h (IQR 1.48–5.03). According to their mRS scores, the patients were divided into two groups with good (*N* = 130, mRS score = 0–2) and poor (*N* = 72, mRS score = 3–6) prognoses. As shown in Table 1, compared with patients with a good prognosis, patients with a poor prognosis were older and had a higher NIHSS score, a higher neutrophil-to-lymphocyte ratio, higher blood glucose, lower triglycerides, lower total cholesterol, and higher HGF levels.

**Table 1:** Clinical characteristics and inflammatory cytokines of AIS patients with favorable and unfavorable outcomes.

**Baseline characteristics**	**All (202)**	**Favorable outcome ** ** (mRS 0–2, *n* = 130)**	**Unfavorable outcome ** ** (mRS 3–6, *n* = 72)**	***p*-value**
Age, y	64.24 ± 13.31	61.92 ± 12.30	68.42 ± 14.11	0.001
Male, *n* (%)	149 (73.76)	98 (75.38)	51 (70.83)	0.481
Time from onset, h	2.95 (1.48–5.03)	2.85 (1.30–4.43)	3.15 (1.80–6.38)	0.108
Baseline systolic BP, mmHg	150 (140–170)	152.5 (140–170)	149 (140–169.5)	0.322
Baseline diastolic BP, mmHg	87.5 (78.0–93.0)	85.0 (77.8–94.0)	90.0 (79.0–92.0)	0.803
Baseline NIHSS	5.0 (3.0–11.0)	4.0 (2.0–6.0)	13.0 (8.3–17.0)	<0.001
Rt-PA administration, *n* (%)	92 (45.54)	68 (52.31)	24 (33.33)	0.009
**Prior risk factors**, ***n*****(%)**				
Hypertension	133 (65.84)	83 (63.85)	50 (69.44)	0.422
Diabetes mellitus	83 (41.09)	47 (36.15)	36 (50.00)	0.055
Coronary heart disease	46 (22.77)	24 (18.46)	22 (30.56)	0.050
Atrial fibrillation	32 (15.84)	11 (8.46)	21 (29.17)	<0.001
**Stroke etiology, n (%)**				
Thrombotic	148 (73.27)	94 (72.31)	54 (75.00)	0.679
Embolic	15 (7.35)	10 (7.63)	5 (6.85)	0.846
Lacunar	39 (19.18)	26 (19.85)	13 (17.81)	0.737
**Clinical parameters, median (IQR)**				
NLR	2.88 (2.01–5.14)	2.55 (1.77–3.99)	4.48 (2.34–8.67)	<0.001
PLT	208.0 (170.8–246.5)	215.5 (175.3–257.0)	196.0 (164.0–229.8)	0.032
Baseline glucose, mmol/L	6.72 (5.71–8.91)	6.32 (5.56–8.04)	8.18 (6.35–11.18)	<0.001
TG, mmol/L	1.50 (0.96–2.48)	1.70 (1.07–2.69)	1.26 (0.79–1.81)	0.007
Total cholesterol, mmol/L	4.55 (3.82–5.44)	4.75 (3.93–5.59)	4.28 (3.60–5.11)	0.018
HDL, mmol/L	1.18 (1.00–1.39)	1.17 (0.97–1.39)	1.23 (1.05–1.39)	0.358
LDL, mmol/L	2.72 (2.05–3.44)	2.79 (2.05–3.62)	2.50 (2.01–3.15)	0.137
**Biomarkers (pg/ml), median (IQR)**				
HGF	101.11 (77.01–132.64)	89.47 (67.61–108.41)	138.96 (99.12–201.03)	<0.001
IL-1β	5.54 (3.46–9.54)	6.30 (3.70–10.69)	4.83 (3.03–8.63)	0.020
IL-5	63.18 (36.62–101.96)	64.49 (44.04–109.02)	57.92 (33.35–92.22)	0.102
IL-10	3.07 (1.89–4.79)	3.07 (1.89–4.68)	3.14 (1.89–4.58)	0.938
IL-16	68.44 (47.29–109.97)	55.86 (43.81–85.38)	89.65 (62.35–147.30)	<0.001

*BP, blood pressure; NIHSS, NIH stroke scale; Rt-PA, recombinant tissue-plasminogen activator; IQR, interquartile range. TG, triglyceride; HDL, high-density lipoprotein; LDL, Low-density lipoprotein*.

### Expression Levels of Plasma HGF and Interleukins in Patients AIS With Unfavorable and Favorable Outcomes at 3 Months

At the 3-month follow-up, 72 (35.64%) patients had experienced the primary unfavorable outcome (Table 2). The clinical variables related to an unfavorable outcome were older age, higher admission NIHSS scores, history of atrial fibrillation, and higher baseline neutrophil-to-lymphocyte ratio. We observed significantly increased baseline HGF (138.96 vs. 89.47, *p* < 0.001) and IL-16 (89.65 vs. 55.86, *p* < 0.001) levels in patients with unfavorable outcome, and baseline IL-1β (4.83 vs. 6.30, *p* = 0.020) levels were decreased in patients with unfavorable outcomes compared with those in patients with favorable outcomes (Table 1).

**Table 2:** Univariable and multivariable logistic regression analyses depicting the associations of the four parameters and other baseline characteristics with unfavorable outcomes.

**Facors**	**Model 1**		**Model 2**	
	**OR (95% CI)**	***p*-value**	**OR (95% CI)**	***p*-value**
Age	1.034 (1.002–1.067)	0.040	1.036 (1.003–1.069)	0.030
NIHSS	1.300 (1.186–1.426)	<0.001	1.309 (1.196–1.432)	<0.001
Diabetes mellitus	2.680 (1.069–6.719)	0.036	2.945 (1.194–7.265)	0.019
Rt-PA administration	0.379 (0.152–0.944)	0.037	0.397 (0.162–0.972)	0.043
**Biomarkers (as continuous variables)**
HGF, 10 pg/ml per increase	1.160 (1.070–1.257)	<0.001	1.178 (1.087–1.277)	<0.001
IL-1β, 1 pg/ml per increase				
IL-5, 10 pg/ml per increase				
IL-10, 1 pg/ml per increase			–	–
IL-16, 10 pg/ml per increase				
**Biomarkers (as categorical variables)**
HGF, ≥117.745 pg/ml			7.606 (3.090–18.726)	<0.001
IL-1β, ≥4.895 pg/ml		–	–	–
IL-5, ≥125.960 pg/ml	–	–	–	–
IL-10, ≥3.660 pg/ml	–	–	–	–
IL-16, ≥60.190 pg/ml	–	–	–	–

*Model 1 was elastic net regression model. Model 2 was adjusted for age, admission NIHSS score, history of diabetes mellitus, and rt-PA treatment*.

### Prognostic Function of Plasma HGF and Interleukin Levels for Poststroke Outcome at 3 Months

We next conducted a multivariable logistic regression analysis to determine whether HGF and its related interleukins could predict the outcome of patients with AIS. After adjusting for clinical variables possibly related to the primary outcome, only elevated HGF level remained significant for the prediction of unfavorable outcomes in binominal multivariate analysis (*p* < 0.001). The multivariable-adjusted OR (95% CI) for HGF (each 10 pg/mL increase) was 1.178 (1.087–1.277) (Table 2). HGF, IL-1β, IL-5, IL-10, and IL-16 levels were then dichotomized using ROC curves. The optimal cutoff values are shown in Table 2. Multivariable logistic regression analyses were then conducted as before using the dichotomized data. Following multivariable analysis, only plasma HGF remained an independent predictor of unfavorable outcome (*p* < 0.001) with a higher adjusted OR of 7.606 (3.090–18.726). None of the other four parameters were independently associated with the primary outcome (Table 2).

### Incremental Predictive Value of Plasma HGF Level for Poor Prognosis of AIS

**Figure 1:**
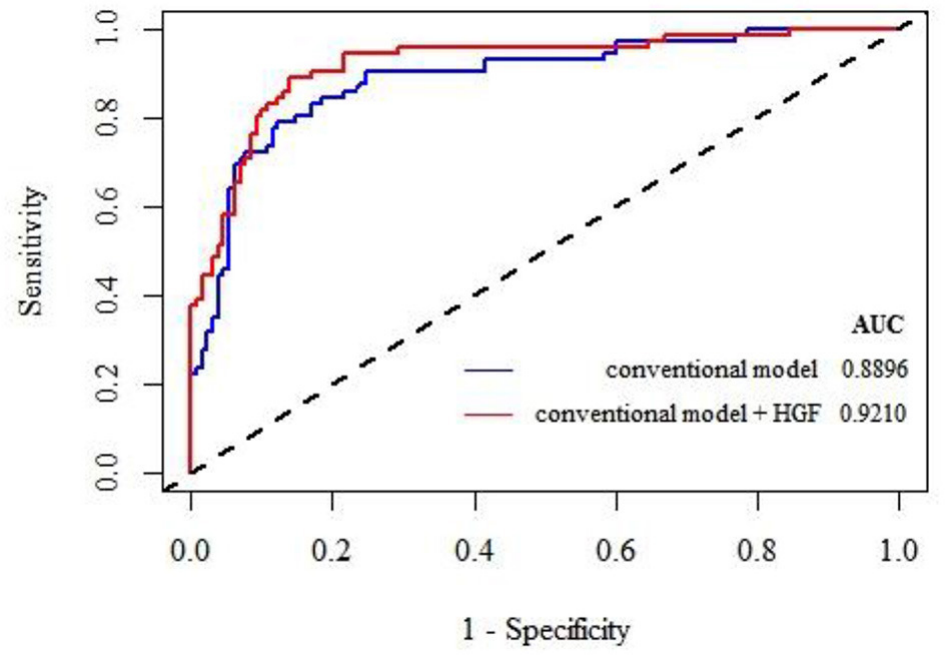
The ROC curves of the two models are drawn to calculate the corresponding AUC value. It can be seen that the AUC value of the traditional model is 0.8896. When HGF is introduced into the model, the AUC value can reach 0.9210, with an increase of 3.1 percentage points.

We then tested whether adding plasma HGF to the conventional model (including age, NIHSS score, diabetes, and t-PA treatment) could improve the predictive ability for unfavorable outcomes in patients with AIS. We calculated the NRI and IDI of the new model compared with the traditional model (Table 3). The results demonstrate that HGF may improve the reclassification of unfavorable outcome (NRI: 31.73%, *p* < 0.001; IDI: 8.71%, *p* < 0.001). We also generated ROC curves of the two models and calculated the corresponding area under the curve (AUC) values (Figure 1). The AUC value of the traditional model was 0.8896, which increased by 3.1 percentage points to 0.9210 when HGF was introduced into the model.

**Table 3:** Reclassification of the primary outcome by plasma HGF in AIS patients.

**Models**	**NRI**		**IDI**	
	**Estimate (95% CI), %**	***p*-value**	**Estimate (95% CI), %**	***p*-value**
Conventional model	Reference	–	Reference	–
Conventional model + HGF	37.13 (19.17–44.29)	<0.001	8.71 (3.75–13.67)	<0.001

*CI, confidence interval; IDI, integrated discrimination index; NRI, net reclassification improvement. The conventional model included factors that are significant in multivariate logistic regression*.

### Plasma HGF Level Was Significantly Higher in Non-survivors of AIS Within 3 Months

**Figure 2:**
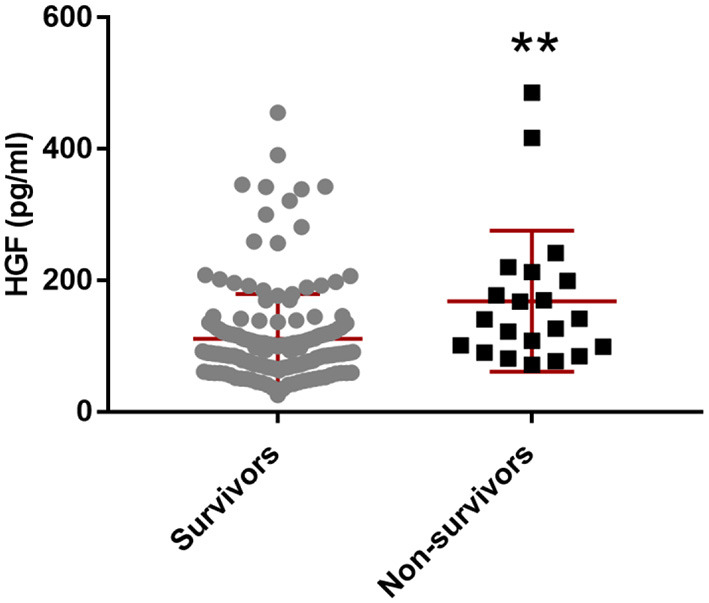
Comparisons of plasma HGF between survivors and non-survivors within 3 months poststroke. The horizontal lines represent median levels and interquartile ranges (IQR). *N* = 181 in survivors, and *n* = 21 in non-survivors. **p < 0.01.

Within 3 months after stroke, 21 patients (10.40%) died. The plasma HGF levels were significantly higher in non-survivors than those in survivors (*p* = 0.0011) (Figure 2). The five biomarkers were dichotomized using ROC curves, and their optimal cutoff values were determined (Table 4). After multiple logistic regression analysis, only plasma HGF was a risk factor for mortality (OR: 3.120, 95% CI: 1.042–9.343, *p* = 0.042) (Table 4).

**Table 4:** Biomarkers and risk of the secondary outcome after AIS.

**Factors**	**Model 1**		**Model 2**	
	**OR (95% CI)**	***p*-value**	**OR (95% CI)**	***p*-value**
NIHSS	1.135 (1.071–1.202)	<0.001	1.115 (1.046–1.188)	0.001
Diabetes mellitus	2.577 (1.017–6.532)	0.046	3.148 (1.091–9.085)	0.034
Coronary heart disease	2.316 (0.895–5.992)	0.083	1.639 (0.528–5.093)	0.561
Atrial fibrillation	2.385 (0.848–6.705)	0.099	0.958 (0.238–3.855)	0.870
Leukocytes number	1.233 (1.087–1.399)	0.001	1.114 (0.942–1.317)	0.204
**Biomarkers (as categorical variables)**
HGF, ≥140.005 pg/ml	6.011 (2.333–15.486)	<0.001	3.120 (1.042–9.343)	0.042
IL-1β, ≥7.035 pg/ml	1.703 (0.686–4.227)	0.251	–	–
IL-5, ≥83.87 pg/ml	1.928 (0.775–4.797)	0.158	–	–
IL-10, ≥2.465 pg/ml	2.163 (0.759–6.164)	0.149	–	–
IL-16, ≥60.190 pg/ml	4.111 (1.331–12.696)	0.014	1.179 (0.475–6.738)	0.413

*Model 1 was an unadjusted logistic regression model. Model 2 was adjusted for admission NIHSS score, history of diabetes mellitus, coronary heart disease, and atrial fibrillation, leukocytes number*.

### HGF Was Closely Associated With Interleukin Levels and Inflammation in Patients With AIS

**Figure 3:**
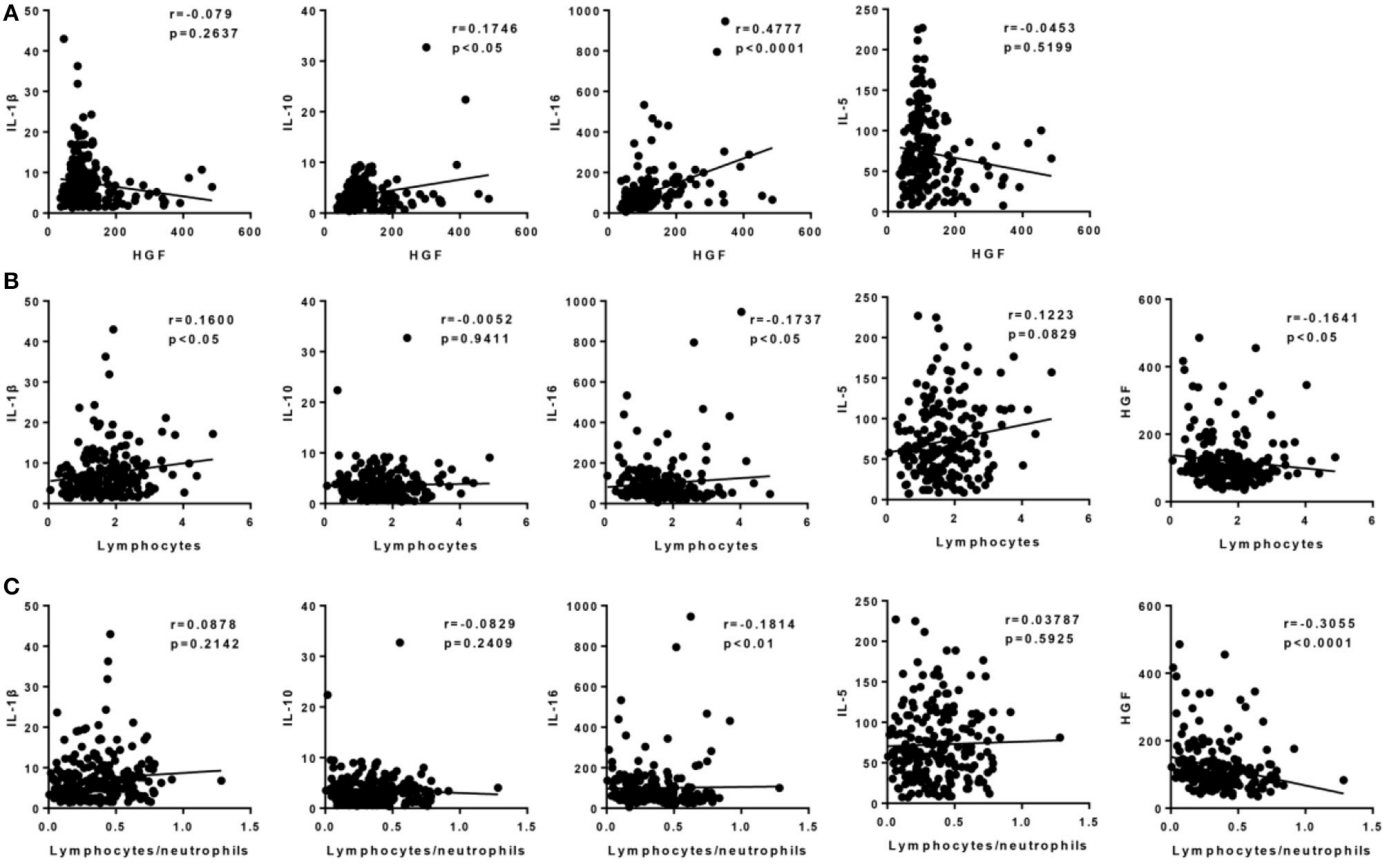
Correlation analyses between circulating HGF and the related interleukins (IL-1β, IL-5, IL-10, and IL-16) in AIS patients. **(A)** Correlation between circulating HGF and other four parameters within 24 h after stroke attack. **(B)** Correlation between the number of circulating lymphocytes and the five parameters within 24 h after stroke attack. **(C)** Correlation between lymphocyte-to-neutrophil ratio and the five parameters within 24 h after stroke attack. *N* = 202.

Correlation analysis to further explore the association of HGF with interleukins and inflammation in patients with AIS revealed that plasma HGF levels were positively related to both IL-10 and IL-16 (Figure 3A, *p* < 0.05). Then, HGF and IL-16 were negatively correlated with lymphocyte number, and IL-1β was positively correlated with lymphocyte number (Figure 3B, *p* < 0.05). Moreover, HGF levels before all interleukins were significantly negatively correlated with the lymphocyte-to-neutrophil ratio (Figure 3C, *p* < 0.0001).

## Discussion

This study investigated the prognostic value of baseline HGF and HGF-associated interleukins in 202 patients with acute-phase AIS and 76 healthy controls. The core findings of this study include (i) elevated plasma HGF levels within 24 h after stroke attack were associated with an increased risk of the primary outcomes at 3 months after stroke in a broader AIS population; moreover, the addition of HGF to a model containing conventional risk factors improved the risk stratification for primary outcomes. (ii) IL-10, IL-1β, IL-5, and IL-16 were not independently associated with unfavorable outcomes in patients with AIS. (iii) HGF was positively correlated with IL-10 and IL-16, and HGF levels before all interleukins and was negatively correlated with lymphocyte-to-neutrophil ratio.

Previously, Zhu et al. reported that serum HGF levels were associated with mortality but not disability at 3 months after ischemic stroke onset (5). However, they included patients within 48 h of symptom onset and excluded patients treated with rt-PA (5). By comparison, we collected blood samples within 24 h and before the patients had received any treatment. Thus, the HGF level could better reflect the pathological changes of AIS without the influence of clinical treatment. We also included patients who received rt-PA therapy for AIS within 4.5 h after symptom onset (19). As rt-PA treatment is a well-accepted strategy, incorporating those patients into this study made our findings more easily generalized. In general, although the inclusion criteria of AIS patients differed somewhat, our results are consistent with those of previous clinical studies reporting that HGF is an independent risk factor for an unfavorable prognosis in patients with AIS.

HGF is a pleiotropic cytokine that can regulate different cellular functions. Delayed recanalization after middle cerebral artery occlusion ameliorated ischemic stroke by inhibiting apoptosis via the HGF/c-Met/STAT3/Bcl-2 pathway in rats (20). However, clinical studies report that circulating HGF level is associated with stroke risk factors involved in endothelial dysfunction, including hypertension, diabetes mellitus, smoking, and age (21). HGF also accelerated the progression of atherosclerosis, and melatonin inhibited macrophage infiltration and promoted plaque stabilization by upregulating the anti-inflammatory HGF/c-Met system in atherosclerotic rabbit ultrasmall superparamagnetic iron oxides (USPIO)-enhanced MRI assessment (22, 23). Similarly, we also found that plasma HGF levels were associated with increased risks of unfavorable outcomes within 3 months in patients with AIS. Collectively, although HGF interventions have shown both good and bad effects in basic research, increased circulating HGF levels were related to the risk factors of stroke and predicted an adverse prognosis of stroke.

HGF was also associated with central and peripheral inflammation in ischemic stroke. HGF suppressed microglial activation and IL-1β expression in rats with ischemic stroke (11). An *in vitro* study also showed that HGF affected the phenotypic shift of macrophages by decreasing the levels of pro-inflammatory IL-1β and promoting the expression of anti-inflammatory IL-10 (12). Our results show the elevation of IL-1β and IL-10 and HGF levels are positively related to IL-10 expression, indicating that HGF may participate in the phenotypic transformation of microglia/macrophages in patients with AIS. Moreover, both HGF and IL-16 were associated with the regulation of Th17, which are important pro-inflammatory cells in AIS. In our study, HGF was strongly positively associated with IL-16 expression, both of which were negatively correlated with lymphocyte number. We speculate that HGF and IL-16 may synergistically regulate the Th17-associated inflammatory process in AIS. HGF levels before all interleukins were substantially negatively correlated with the lymphocyte-to-neutrophil ratio as well as unfavorable outcomes in patients with AIS. Therefore, we inferred that HGF was involved in multiple inflammatory responses in AIS and that interventions involving HGF may be a therapeutic strategy in AIS.

However, our study has some limitations. First, it lacks data on infarct volume in CT or MRI scans. Patients who met the criterion of intravenous therapy undergo CT scans to exclude cerebral hemorrhage and should be infused with thrombolysis drugs as early as possible (24). Thus, nearly half of the patients in our study did not undergo a premorbid MRI scan. However, infarcts are not obvious on early CT scans, and the severity of neurological prognosis is not always proportional to the size of infarct volume; hence, it is reasonable that we did not include infarct volume in our study. Second, our study was performed in a single center, and the sample size is somewhat small, which limits the generalizability of our results. Further testing in separate cohorts with larger sample sizes in multiple centers is needed to verify our findings. Third, the biomarkers included in this study were only examined once at admission. As the inflammatory process is dynamic after AIS, it is essential to monitor the changes of these biomarkers in further studies. Nevertheless, the present study extends the knowledge and clinical application of acute-phase circulating HGF levels in a broader AIS population.

In conclusion, our study results demonstrate that increased plasma HGF levels within 24 h were associated with unfavorable prognosis and mortality at 3 months after AIS. Adding plasma HGF to established risk factors substantially improves the risk prediction for an unfavorable prognosis in patients with AIS patients; thus, the results of the present study extend the prognostic significance of HGF in patients with AIS administered t-PA treatment within 24 h poststroke. In addition, we find that plasma HGF may be a node for phenotype transformation of inflammatory cells in AIS. However, our findings also have limitations, including the fact that, due to the characteristics of the prediction (its bidirectionality), we cannot determine which is the cause and which is the effect. However, this link provides a thought-provoking example of how apparently different biological properties could interact to determine a unique abnormal condition of disease. If this association is verified, interventions including HGF may offer a new, promising immunomodulatory therapeutic target for AIS.

## Data Availability Statement

The original contributions presented in the study are included in the article/supplementary material, further inquiries can be directed to the corresponding author/s.

## Ethics Statement

The studies involving human participants were reviewed and approved by Ethics Committee of Xuanwu Hospital, Capital Medical University. The patients/participants provided their written informed consent to participate in this study.

## Author Contributions

FL and PL prepared the study protocol, collected, analyzed, interpreted the data, and prepared the manuscript. YH, LL, SZ, ZY, RW, ZT, ZH, JF, and YZ collected the data. FL and PL performed the cytometric assay and analyzed the data. HZ and YL prepared the study protocol, analyzed and interpreted the data, and supervised the study. All authors contributed to the article and approved the submitted version.

## Conflict of Interest

The authors declare that the research was conducted in the absence of any commercial or financial relationships that could be construed as a potential conflict of interest.

## Publisher's Note

All claims expressed in this article are solely those of the authors and do not necessarily represent those of their affiliated organizations, or those of the publisher, the editors and the reviewers. Any product that may be evaluated in this article, or claim that may be made by its manufacturer, is not guaranteed or endorsed by the publisher.
